# Supplementary material for: Mapping the evolution of fertility support policies in China: A content and instrumental analysis
Source: PLoS One. 2025 Oct 9;20(10):e0332137. doi: 10.1371/journal.pone.0332137 (PMC12510515; doi:10.1371/journal.pone.0332137)
Supplement: S1 Appendix — (ZIP) [file pone.0332137.s001.zip › S1 Appendix. 226 original policy documents/226-财政部、国家计划生育委员会关于颁发《计划生育事业单位财务制度》的通知(FBM-CLI-4-20569).doc]

财政部、国家计划生育委员会关于颁发《计划生育事业单位财务制度》的通知

**发布部门：** [财政部](https://www.pkulaw.com/law/chl?Aggs.IssueDepartment=60210) [国家计划生育委员会(已更名)](https://www.pkulaw.com/law/chl?Aggs.IssueDepartment=60314) [机构沿革](javascript:void(0))

**发文字号：**财文字〔1997〕274号

**发布日期：**1997.06.09

**实施日期：**1997.06.09

**时效性：** [失效](https://www.pkulaw.com/law/chl?Aggs.TimelinessDic=02)

**效力级别：** [部门工作文件](https://www.pkulaw.com/law/chl?Aggs.EffectivenessDic=XE0303)

**法规类别：** [行政事业财务管理](https://www.pkulaw.com/law/chl?Aggs.Category=03305)

**修改依据：**[财政部、人口计生委关于印发《人口和计划生育事业单位财务制度》的通知(2012修订)](javascript:TMP(193183,0,'chl'))

**失效依据：**[财政部关于公布废止和失效的财政规章和规范性文件目录(第十二批)的决定](javascript:TMP(279454,0,'chl'))

　　　　　　　　财政部　国家计划生育委员会关于颁发《计划
　　　　　　　　　　　生育事业单位财务制度》的通知
　　　　　 （１９９７年６月９日　财文字［１９９７］２７４号）

各省、自治区、直辖市、计划单列市财政厅（局）、计划生育委员会：
　　为了更好地规范计划生育事业单位财务行为，加强财务管理，促进计划生育事业的发展，根据《[事业单位财务规则](https://www.pkulaw.com/chl/6d528f7681f9bd07bdfb.html)》，结合计划生育事业单位自身特点，我们制定了《计划生育事业单位财务制度》。现颁发给你们，请遵照执行。
　　附件：计划生育事业单位财务制度
附件：
　　　　　　　　　　　　计划生育事业单位财务制度

　第一章　总则

　　**第一条**　为了规范计划生育事业单位的财务行为，加强财务管理，提高资金使用效益，保障计划生育事业的健康发展和计划生育基本国策的贯彻执行，根据《[事业单位财务规则](https://www.pkulaw.com/chl/6d528f7681f9bd07bdfb.html)》，结合计划生育事业单位的实际情况，制定本制度。

　　**第二条**　本制度适用于各级政府计划生育行政主管部门（以下简称主管部门）所属的各级各类计划生育事业单位的财务活动。

　　**第三条**　计划生育事业单位财务管理的基本原则是：遵守国家有关法律、法规和财务规章制度；坚持勤俭办事的方针；正确处理事业发展需要和资金供给的关系，社会效益和经济效益的关系，国家、集体和个人三者利益的关系。

　　**第四条**　计划生育事业单位财务管理的主要任务是：合理编制单位预算；依法组织收入，合理安排支出；建立健全财务制度，加强经济核算，提高资金使用效益；加强国有资产管理，防止国有资产流失；如实反映单位财务状况；参与单位的重大经济决策和对外签订经济合同等事项，对单位经济活动进行控制和监督。

　　**第五条**　计划生育事业单位的财务工作，应接受财政、审计、税务等有关部门和主管部门的指导、检查和监督。

　　**第六条**　计划生育事业单位的全部财务活动在单位负责人领导下，由单位财务部门统一管理。

　第二章　单位预算管理

　　**第七条**　单位预算是指计划生育事业单位根据事业发展计划和任务编制的年度财务收支计划。计划生育事业单位预算由收入预算和支出预算组成。

　　**第八条**　国家对计划生育事业单位实行核定收支、定额或者定项补助、超支不补、结余留用的预算管理办法。定额或者定项补助标准根据计划生育事业特点、事业发展计划、单位收支状况以及国家财政政策和财务可能确定。定额或者定项补助可以为零。
　　少数非财政补助收入大于支出较多的计划生育事业单位，可实行收入上缴办法。具体办法由财政部门会同主管部门制定。

　　**第九条**　预算编制原则：
　　（一）根据国家有关方针、政策，国家有关法律、法规和规章制度，以及计划生育事业计划编制单位预算。
　　（二）坚持实事求是的原则，既要考虑单位的需要，又要考虑国家财务的可能，保证重点、兼顾一般。
　　（三）坚持以收定支、收支平衡的原则，单位预算应自求平衡，不得编制赤字预算。
　　（四）坚持艰苦奋斗、勤俭节约的原则，挖掘内部潜力，努力增收节支，提高资金使用效益。
　　（五）坚持严格划清经费渠道的原则。事业经费与基本建设投资不得相互挤占和挪用，并根据有关规定，分别编制预算。
　　（六）坚持完整性和统一性原则。计划生育事业单位必须将全部财务收支项目在预算中予以反映，并按照国家预算表格和统一的口径、程序及计算依据编制单位预算。

　　**第十条**　预算编制程序：
　　计划生育事业单位根据年度事业计划，提出预算建议数，经主管部门审核汇总报财政部门核定。单位根据财政部门下达的预算控制数编制预算，由主管部门汇总报财政部门审核批复后执行。
　　预算报送时间按财政部门的规定执行。

　　**第十一条**　预算编制方法：
　　（一）收入预算包括财政补助收入、上级补助收入、事业收入、经营收入、附属单位上缴收入和其他收入。收入预算根据事业计划，参照近年收入情况，并考虑预算年度可能出现的收入增减因素编制。
　　（二）支出预算包括事业支出、经营支出、自筹基本建设支出、对附属单位补助支出和上缴上级支出。支出预算根据事业发展需要与财力可能测算编制。
　　１．事业支出包括基本工资、其他工资、补助工资、职工福利费、社会保障费、助学金、公务费、业务费、设置购置费、修缮费和其他费用。其中；基本工资、其他工资、补助工资、职工福利费、社会保障费和助学金，根据单位人员编制、劳动工资计划及其他有关规定进行编制；公务费按照人员编制、定额标准或近年来支出的情况进行测算编制；业务费按工作任务和定额编制，无法确定定额的，按近年开支情况测算编制；设备购置费按主管部门批准的设备购置计划和市场价格进行编制；修缮费按维修定额或实际情况进行测算编制；其他费用测算编制。
　　２．经营支出目级科目与事业支出目级科目相同。预算编制方法比照事业支出编制方法办理。
　　３．自筹基本建设支出按照本年用财政补助收入以外资金安排自筹基本建设项目支出数额编制。
　　４．对附属单位补助支出按主管部门批准的数额编制。
　　５．上缴上级支出按规定的比例或数额编制。
　　（三）单位预算必须附有预算编制说明书。

　　**第十二条**　预算调整：
　　计划生育事业单位预算在执行过程中，财政补助收入、从财政专户核拨的预算外资金一般不予调整。但是，上级下达的事业计划有大的调整，或者根据国家有关政策增加或减少支出，对预算执行影响较大时，单位需报请主管部门或财政部门调整预算；非财政补助收入部分需要调增调减的，由单位自行调整并报主管部门和财政部门备案。
　　收入预算调整后相应调增调减支出预算。

　第三章　收入管理

　　**第十三条**　收入是指计划生育事业单位为开展业务及其他活动依法取得的非偿还性资金。

　　**第十四条**　计划生育事业单位的收入包括：
　　（一）财政补助收入，即计划生育事业单位通过主管部门、上级单位或直接从财政部门取得的计划生育事业费，包括经常性经费和专项资金。
　　（二）上级补助收入，即计划生育事业单位从主管部门或上级单位取得的各种非财政补助收入。
　　（三）事业收入，即计划生育事业单位开展专业业务活动及其辅助活动取得的收入，其中：按照国家有关规定应当上缴财政的资金和应当缴入财政专户的预算外资金不计入事业收入；从财政专户核拨的预算外资金和部分经核准不上缴财政专户管理的预算外资金收入计入事业收入。
　　（四）经营收入，即计划生育事业单位在专业业务活动及其辅助活动之外开展非独立核算经营活动取得的收入。
　　（五）附属单位上缴收入，即计划生育事业单位附属独立核算单位按照有关规定上缴的收入。
　　（六）其他收入，即上述规定范围以外的各项收入，包括投资收益、利息收入、捐赠收入等。

　　**第十五条**　事业收入包括：
　　（一）技术服务收入，即计划生育事业单位开展计划生育优生优育咨询指导，避孕节育手术、接生等医疗服务活动取得的收入。
　　（二）病残儿鉴定收入，即计划生育事业单位对病残儿进行鉴定所取得的收入。
　　（三）代培进修收入，即计划生育事业单位为其他单位代培计划生育工作人员取得的收入。
　　（四）宣传品制作收入，即计划生育事业单位制作计划生育文字和音像等宣传品取得的收入。
　　（五）无形资产转让收入，即计划生育事业单位按照国家有关规定，经过中介机构评估后转让无形资产取得的收入。
　　（六）其他事业收入，即计划生育事业单位开展专业业务活动及其辅助活动所取得的除上述各项收入以外的收入。
　　计划生育事业单位上述六项收入中，属于从财政专户领拨的预算外资金以及经财政部门核准不上缴财政专户管理的预算外资金，作为单位的预算外收入计入事业收入，同时在预算外资金收支计划表中反映。

　　**第十六条**　经营收入包括：
　　（一）销售收入，即计划生育事业单位非独立核算部门销售商品取得的收入。
　　（二）经营服务收入，即计划生育事业单位非独立核算部门对外提供经营服务取得的收入。
　　（三）租赁收入，即计划生育事业单位出租房屋、场地和设备取得的收入。
　　（四）其他经营收入，即计划生育事业单位在专业业务活动及辅助活动之外取得的除上述各项收入以外的收入。

　　**第十七条**　收入管理的要求：
　　（一）计划生育事业单位应当在国家政策允许的范围内，依法组织收入，坚持把社会效益放在首位，同时注重经济效益。
　　（二）计划生育事业单位必须使用财政部门和税务部门统一印制的票据，并建立健全各种专用收款收据、销售发票等票据的管理制度。
　　（三）计划生育事业单位必须严格执行国家批准的收费项目和收费标准，不得擅自设立收费项目，自定收费标准。
　　（四）计划生育事业单位应当按照规定加强帐户的统一管理，收入要及时入帐，防止流失。
　　（五）计划生育事业单位的各项收入必须全部纳入单位预算，统一核算，统一管理。

　第四章　支出管理

　　**第十八条**　支出是计划生育事业单位开展业务及其他活动发生的资金耗费和损失。

　　**第十九条**　计划生育事业单位支出包括事业支出、经营支出、自筹基本建设支出、对附属单位补助支出和上缴上级支出。
　　（一）事业支出，即计划生育事业单位开展专业业务活动及其辅助活动发生的支出，包括基本工资、其他工资、补助工资、职工福利费、社会保障费、助学金、公务费、业务费、设备购置费、修缮费和其他费用。
　　（二）经营支出，即计划生育事业单位在专业业务活动及其辅助活动之外开展非独立核算经营活动发生的支出。
　　（三）自筹基本建设支出，即事业单位用财政补助收入以外的资金安排的自筹基本建设发生的支出。事业单位自筹资金安排基本建设，应先落实资金来源，并按审批权限，报经有关部门列入基本建设计划。事业单位应在保证正常事业支出需要，保证正常预算平衡的基础上，统筹安排自筹基本建设支出，并报主管部门和财政部门核批。核定的自筹基本建设资金纳入基本建设财务管理。
　　（四）对附属单位补助支出，即计划生育事业单位用财政补助收入之外的收入对附属单位补助发生的支出。
　　（五）上缴上级支出，即实行收入上缴办法的计划生育事业单位按照规定的定额或者比例上缴上级单位的支出。

　　**第二十条**　计划生育事业单位在开展非独立核算经营活动中，应当正确归集实际发生的各项费用数，不能归集的，应当按照规定的比例合理分摊。经营支出应与经营收入配比。

　　**第二十一条**　计划生育事业单位应当建立健全支出管理制度。各项支出应在单位负责人的领导下，由单位财务部门在财政部门和主管部门核定的预算指标之内统一掌握使用。单位各业务部门的开支，应当事先提出使用计划交单位财务部门审核后执行。

　　**第二十二条**　计划生育事业单位应当严格执行国家规定的开支范围及开支标准；没有统一规定的，由计划生育事业单位做出规定，报主管部门和财政部门备案。计划生育事业单位的规定违反国家法律、法规和政策的，主管部门和财政部门应当责令其改正。

　　**第二十三条**　计划生育事业单位从财政部门和主管部门取得的有指定项目和用途并且要求单独核算的专项资金，应当按照要求定额向财政部门和主管部门报送专项资金使用情况；项目完成后，应当报送专项资金支出决算和使用效果的书面报告，接受财政部门和主管部门的检查和验收。

　　**第二十四条**　国家计划生育委员会和财政部联合拨付地方的计划生育事业补助费和计划生育避孕药具专款的管理和使用，分别执行《计划生育事业补助费管理办法》和《计划生育避孕药具专款管理办法》。

　　**第二十五条**　为了加强支出管理，提高经济核算水平，有条件的计划生育事业单位应根据开展业务及其他活动的实际需要，实行内部成本核算办法。其支出根据成本费用开支范围可以划分为直接费用、间接费用和期间费用。
　　（一）直接费用是指直接从事计划生育技术服务、药具贮藏、运输和发放、宣传品制作等业务活动和非独立核算生产经营活动所发生的费用。直接费用计入产品成本中。
　　（二）间接费用是指计划生育事业单位内部各业务部门为组织和管理计划生育业务和非独立核算生产经营活动所发生的费用。间接费用按一定比例计入成本中。
　　（三）期间费用是指计划生育事业单位内部行政后勤管理部门发生的各项费用。期间费用计入当期结余。

　　**第二十六条**　实行内部成本核算办法的范围：
　　（一）非财政补助收入能够基本满足正常支出的计划生育避孕药具供应站、药具管理站、计划生育服务站等单位。
　　（二）主管部门和计划生育事业单位附属的非独立核算的商品销售部、餐饮部等。

　　**第二十七条**　实行内部成本核算办法的单位、单位内部实行成本核算的部门，其成本费用须按支出用途分别归集到单位事业支出和经营支出的相应科目中去。

　　**第二十八条**　计划生育事业单位的基本建设支出、对外投资支出以及国家规定不得列入成本费用的其他支出，不得计入成本费用。

　第五章　结余及其分配

　　**第二十九条**　结余是计划生育事业单位年度收入与支出相抵后的余额。

　　**第三十条**　计划生育事业单位的结余（不含实行预算外资金结余上缴办法的预算外资金结余），除专项资金按照规定结转下一年度继续使用外，可以按照规定提取职工福利基金，剩余部分作为事业基金，用于弥补以后年度收支差额。国家另有规定的，从其规定。
　　计划生育事业单位的经营收支结余应当单独反映。经营收支结余先按照国家有关规定弥补以前年度经营收支发生的亏损，其余部分并入单位的结余中进行分配。

　第六章　专用基金管理

　　**第三十一条**　专用基金是指计划生育事业单位按照规定提取或设置的有专门用途的资金。

　　**第三十二条**　专用基金包括：
　　（一）修购基金，即按照事业收入和经营收入的一定比例提取，在修缮费和设备购置费中列支（各列５０％），用于计划生育事业单位固定资产维修、购置的资金。计划生育事业单位的固定资产变价收入和有偿调拨收入，除国家另有规定者外，直接转入修购基金。
　　（二）职工福利基金，即按照结余的一定比例提取转入，用于单位职工的集体福利设施、集体福利待遇等的资金。
　　（三）医疗基金，即未纳入公费医疗经费开支范围的单位，按照当地财政部门规定的公费医疗经费开支标准提取，并参照公费医疗制度有关规定用于职工公费医疗开支的资金。
　　（四）其他基金，即按照其他有关规定提取和设置的专用基金。

　　**第三十三条**　各项基金的提取比例和管理办法，国家有统一规定的，按统一规定执行，没有统一规定的由主管部门会同同级财政部门确定。

　第七章　资产管理

　　**第三十四条**　资产是计划生育事业单位占有或者使用的能以货币计量的经济资源，包括各种财产、债权和其他权利。

　　**第三十五条**　计划生育事业单位的资产包括流动资产、固定资产、无形资产和对外投资等。

　　**第三十六条**　流动资产是指可以在一年内变现或者耗用的资产，包括现金、各种存款、应收款项、预付款项和存货等。

　　**第三十七条**　这行独立核算的计划生育事业单位必须按照国家有关规定在银行开设存款帐户，严格遵守银行制度，接受银行监督。
　　在银行开户必须报主管部门批准。一般由单位财务部门开设帐户，单位内部其他非独立核算部门不得另设帐户。银行存款帐户只限于本单位使用，不得出租、出借或转让给外单位或个人使用。

　　**第三十八条**　计划生育事业单位对应收款项和预付款项要按时清理结算，不得长期挂帐。对确定实无法收回的预付款，要查明原因，分清责任，报经主管部门批准后核销；数额较大的，须报请同级财政部门批准后核销。

　　**第三十九条**　存货是指计划生育事业单位在开展业务及其他活动中为耗用而储存的资产，包括材料、燃料、包装物、避孕药具、低值易耗品等。
　　（一）计划生育事业单位应当建立健全存货管理制度。单位财产物资管理部门应指定专人负责，严格收发手续，完善存货验收、进出库和保管制度，防止丢失、损坏、变质。
　　（二）单位财产物资管理部门应当根据工作需要编制采购计划，经单位财务部门审核报单位负责人批准后进行采购。
　　（三）单位财产物资管理部门应当建立材料明细帐，定期与财务部门的材料总帐进行核对，做到帐帐相符、帐实相符。
　　（四）制定材料储备定额和主要材料消耗定额。
　　（五）计划生育事业单位应当对存货进行定期或不定期的清查盘点，保证帐实相符。存货盘盈、盘亏应及时调帐。

　　**第四十条**　固定资产是指一般设备单位价值在５００元以上，专用设备单位价值在８００元以上，使用期限在一年以上，并在使用过程中基本保持原有物质形态的资产。单位价值虽未达到规定标准，但耐用时间在一年以上的大批同类物资，作为固定资产管理。
　　固定资产一般分为六类：房屋和建筑物；专用设备；一般设备；文物和陈列品；图书；其他固定资产。
　　主管部门可以根据本系统具体情况制定各类固定资产明细目录。

　　**第四十一条**　计划生育事业单位要加强固定资产管理：
　　（一）建立健全固定资产管理制度，加强固定资产维护和保养，制定操作规程，建立技术档案和使用情况报告制度。
　　（二）购建和调入的固定资产，由单位财产物资管理部门负责验收，单位财务部门参与验收。购进贵重仪器等专业设备和新建的房屋及建筑物竣工时，应有专业技术人员参加验收。经验收后的固定资产要及时入帐并交付使用。
　　（三）接受捐赠的固定资产，应当按照市场价格和新旧程度估价入帐，或根据捐赠时提供的有关凭据确定固定资产的价值。接受捐赠固定资产时发生的各项费用，计入固定资产原值。
　　（四）计划生育事业单位固定资产报废和转让，一般经本单位负责人批准后核销。大型、精密贵重设备、仪器报废和转让，应当经过有关部门鉴定，报主管部门或国有资产管理部门、财政部门批准。具体审批权限由同级财政部门会同国有资产管理部门规定。
　　（五）固定资产的变价收入，除国家另有规定者外转入修购基金。
　　（六）计划生育事业单位应当定期或者不定期对固定资产清查盘点。年度终了前必须进行一次全面的清查盘点，做到帐、卡、物相符。对于盘盈、盘亏的固定资产应当及时按照规定处理。

　　**第四十二条**　计划生育避孕药具的管理按照本制度的《药具管理站实施细则》执行。

　　**第四十三条**　无形资产是指不具有实物形态而能为使用者提供某种权利的资产，包括专利权、商标权、著作权、土地使用权、非专利技术、商誉以及其他财产权利等。
　　（一）计划生育事业单位应当对本单位无形资产的评估确认、开发、保护、使用和转让进行管理。
　　（二）无形资产的有效使用期限按照国家有关法律、法规确定。
　　（三）计划生育事业单位转让无形资产，应当按照有关规定进行资产评估，取得的收入除国家另有规定者外计入事业收入。计划生育事业单位取得无形资产发生的支出应计入事业支出。

　　**第四十四条**　对外投资是计划生育事业单位利用货币资金、实物、无形资产等方式向其他单位的投资。
　　（一）计划生育事业单位对外投资，应当按照国家有关规定报经主管部门、国有资产管理部门和财政部门批准。
　　（二）以实物、无形资产对外投资的，应当按照国家有关规定进行资产评估。
　　（三）对外投资必须进行充分的技术和经济效益论证，保证国有资产的完整和增值。

　第八章　负债管理

　　**第四十五条**　负债是计划生育事业单位所承担的能以货币计量，需要以资产或劳务偿还的债务。

　　**第四十六条**　计划生育事业单位的负债包括借入款项、应付款项、暂存款项、应缴款项等。
　　（一）借入款是指计划生育事业单位开展各项活动向财政部门、主管部门、上级单位、金融机构和其他单位借入的款项。
　　（二）应付款项和暂存款项是指计划生育事业单位按照有关规定和要求，应付未付的各种款项。
　　（三）应缴款项是指计划生育事业单位收取的应当上缴财政预算的资金和应当上缴财政专户的预算外资金、应缴税金以及其他按照国家有关规定应上缴的款项。

　　**第四十七条**　计划生育事业单位应对不同性质的负债分别管理，及时清理并按照规定办理结算，保证各项负债在规定期限内归还。

　第九章　事业单位清算

　　**第四十八条**　计划生育事业单位发生划转撤并时，应当进行清算。

　　**第四十九条**　计划生育事业单位清算，应当在主管部门和财政部门、国有资产管理部门的监督指导下，对单位的财产、债权、债务等进行全面清查，编制资产负债表、财产目录和债权、债务清单，提出财产作价依据和债权、债务处理办法，做好国有资产的移交、接收、划转和管理工作，并妥善处理各项遗留问题。

　　**第五十条**　计划生育事业单位清算结束后，经主管部门审核并报国有资产管理部门和财政部门批准，其资产分别按下列办法处理：
　　（一）因隶属关系改变，成建制划转的计划生育事业单位，其全部资产无偿移交，并相应划转事业经费指标。
　　（二）转为企业管理的计划生育事业单位，全部资产扣除负债后，转作国家资本金。
　　（三）撤销的计划生育事业单位，全部资产由主管部门和财政部门核准处理。
　　（四）合并的计划生育事业单位，全部资产移交接收单位或新组建单位。合并后多余的国有资产，由主管部门和财政部门核准后处理。

　第十章　财务报告和财务分析

　　**第五十一条**　财务报告是反映计划生育事业单位一定时期财务状况和经营成果的总结性书面文件。
　　计划生育事业单位应当按照财政部门和主管部门的规定和要求，定期编制财务报告，做到内容完整、数据真实、计算准确、说明符合实际情况，并定期向主管部门和财政部门以及其他有关的报表使用者提供财务报告。

　　**第五十二条**　计划生育事业单位报送的年度财务报告包括资产负债表、收支情况表、有关附表以及财务情况说明书。

　　**第五十三条**　财务情况说明书，主要说明计划生育事业发展状况、事业单位收入及其支出、结余及其分配、资产负债变动情况、对本期或者下期财务情况发生重大影响的事项，以及需要说明的其他事项。

　　**第五十四条**　计划生育事业单位财务分析的内容包括预算执行、资产使用、支出状况、财务管理和事业效果等。

　　**第五十五条**　财务分析指标为财务指标和业务指标两类。
　　（一）财务指标包括：经费自给率、人员支出与公用支出分别占事业支出的比率、资产负债率、预算完成率、事业收入占总收入的比率、、经营收入占总收入的比率、事业收入增长率、经营收入增长率、避孕药具库存量及库存结构等。
　　（二）业务指标包括：宣传品制作品种、宣传品发行量、宣传品进村入户率、计划生育培训人次、避孕药具应用率、药具使用有效率、四项手术例数及占本地区手术总量的比重等。
　　除上述指标外，单位可以根据本单位业务特点确定财务分析指标。

　第十一章　附则

　　**第五十六条**　国家对计划生育事业单位基本建设投资的财务管理，依据有关规定办理。

　　**第五十七条**　接受国家经常性资助的非国有计划生育事业单位，依照本制度执行；其他计划生育事业单位可以参照本制度执行。

　　**第五十八条**　下列事业单位或者事业单位的特定项目，执行《[企业财务通则](https://www.pkulaw.com/chl/f4fad3c62e7d2184bdfb.html)》和同行业或者相近行业财务制度，不执行本制度：
　　（一）纳入企业财务管理体系的计划生育事业单位和事业单位附属独立核算的生产经营单位；
　　（二）计划生育事业单位接受外单位要求投资回报的经营项目；
　　（三）经主管部门和财政部门批准的具备条件的计划生育事业单位。

　　**第五十九条**　主管部门所属的计划生育科学研究单位和学校，执行同行业事业单位财务制度。

　　**第六十条**　计划外生育费收入的管理和使用，执行《[计划外生育费管理办法](https://www.pkulaw.com/chl/569d7e8f3e12d247bdfb.html)》。

　　**第六十一条**　各省、自治区、直辖市及计划单列市的计划生育主管部门和财政部门，可根据本制度，结合本地区实际情况，制定补充规定，报财政部、国家计划生育委员会备案。计划生育事业单位可按照本制度，根据单位实际情况，制定具体规定，并报主管部门备案。

　　**第六十二条**　本制度由财政部负责修订和解释。

　　**第六十三条**　本制度自１９９７年１月１日起实施。

　　附：财务分析指标
附：
　　　　　　　　　　　　　　　财务分析指标
　　　　　　　　事业收入＋经营收入＋附属单位上缴收入＋其他收入
　　经费自给率＝－－－－－－－－－－－－－－－－－－－－－－－×１００％
　　　　　　　　　　　　　　　事业支出＋经营支出
　　人员支出占事业支出比率
　　　　基本工资＋其他工资＋补助工资＋职工福利费＋社会保障费
　　　＝－－－－－－－－－－－－－－－－－－－－－－－－－－×１００％
　　　　　　　　　　　　　事业支出
　　公用支出占事业支出比率
　　　　公务费＋设备购置费＋修缮费＋业务费＋其他费用
　　　＝－－－－－－－－－－－－－－－－－－－－－－×１００％
　　　　　　　　　　　事业支出
　　　　　　　　负债总额
　　资产负债率＝－－－－×１００％
　　　　　　　　资产总额
　　　　　　　　当年预算完成数
　　预算完成率＝－－－－－－－×１００％
　　　　　　　　当年预算计划数
　　　　　　　　　　　　　　事业收入
　　事业收入占总收入的比率＝－－－－×１００％
　　　　　　　　　　　　　　 总收入
　　　　　　　　　　　　　　经营收入
　　经营收入占总收入的比率＝－－－－×１００％
　　　　　　　　　　　　　　 总收入
　　　　　　　　　　　当年事业收入
　　事业收入增长率＝（－－－－－－—１）×１００％
　　　　　　　　　　　上年事业收入
　　　　　　　　　　　当年经营收入
　　经营收入增长率＝（－－－－－－—１）×１００％
　　　　　　　　　　　上年经营收入
　　　　　　　　　　　有宣传品的户数
　　宣传品进村入户率＝－－－－－－－×１００％
　　　　　　　　　　　　村总户数
　　　　　　　　　　已使用药具人数
　　避孕药具应用率＝－－－－－－－×１００％
　　　　　　　　　　应采用药具人数
　　　　　　　　　　使用药具有效人数
　　药具使用有效率＝－－－－－－－－×１００％
　　　　　　　　　　 已采用药具人数

© 北大法宝：（[www.pkulaw.com](https://www.pkulaw.com/)) 专业提供法律信息、法学知识和法律软件领域各类解决方案。北大法宝为您提供丰富的参考资料，正式引用法规条文时请与标准文本核对。
欢迎查看所有[产品和服务](https://www.pkulaw.com/helps/2.html)。法宝快讯：[如何快速找到您需要的检索结果？](https://www.pkulaw.com/helps/70.html)[法宝](https://www.pkulaw.com/helps/69.html)V6有何新特色？

扫描二维码阅读原文

**原文链接：**<https://www.pkulaw.com/chl/6a9e666d860aa87bbdfb.html>
